# Overexpression of *SmMYC2* Increases the Production of Phenolic Acids in *Salvia miltiorrhiza*

**DOI:** 10.3389/fpls.2017.01804

**Published:** 2017-10-18

**Authors:** Na Yang, Wenping Zhou, Jiao Su, Xiaofan Wang, Lin Li, Liru Wang, Xiaoyan Cao, Zhezhi Wang

**Affiliations:** Key Laboratory of the Ministry of Education for Medicinal Resources and Natural Pharmaceutical Chemistry, National Engineering Laboratory for Resource Development of Endangered Crude Drugs in Northwest of China, Shaanxi Normal University, Xi’an, China

**Keywords:** *SmMYC2*, *Salvia miltiorrhiza*, secondary metabolism, salvianolic acid B, tanshinones, transcriptome

## Abstract

MYC2 is a core transcription factor in the plant response to jasmonates. It also functions in secondary metabolism and various processes for growth and development. However, the knowledge about its role in *Salvia miltiorrhiza* is still very limited. We determined that the biosynthesis of salvianolic acid B (Sal B) was strongly induced in 2-month-old transgenic plants that over-expressed *SmMYC2*. In the roots of transgenic line 12 that over-expressed *SmMYC2* (OEM-12), the Sal B concentration was as high as 5.95 ± 0.07 mg g^-1^, a level that was 1.88-fold higher than that in control plants that had been transformed with an empty vector. Neither tanshinone IIA nor cryptotanshinone was detected by high-performance liquid chromatography in any of the genotypes. Global transcriptomic analysis using RNA sequencing revealed that most enzyme-encoding genes for the phenylpropanoid biosynthesis pathway were up-regulated in the overexpression lines. Furthermore, both the phenylalanine and tyrosine biosynthesis pathways were activated in those transgenics. Our data demonstrate that overexpression of *SmMYC2* promotes the production of phenolic acids by simultaneously activating both primary and secondary pathways for metabolism in *S. miltiorrhiza*.

## Introduction

*Salvia miltiorrhiza* Bunge is a very useful medicinal plant and has been applied to treat various maladies, especially coronary and cerebrovascular diseases (Chun-Yan et al., 2015). As one of the best-known Chinese traditional herbs, its root has been clinically used for more than 2000 years (Guo et al., 2014). According to pharmacological investigations, active ingredients in *S. miltiorrhiza* are classified as lipophilic diterpene quinone pigments, generally known as tanshinones (Liao et al., 2009) and hydrophilic phenolic acids, which are mainly rosmarinic acid (RA) and its derivative salvianolic acid B (Sal B) (Huang et al., 2008; Gu et al., 2012). Among the phenolic acids, Sal B is considered the most important active content when *S. miltiorrhiza* is extracted with water in a traditional method. Designated as a marker component of *S. miltiorrhiza* in the official Chinese Pharmacopeia, Sal B appears to offer protection against hepatic, neural, and cardiovascular diseases, as well as certain cancers (Ho, 2011). Because of the economic value of Sal B, exogenous transcription factors (TFs) have been used with genetic engineering to enhance its production in *S. miltiorrhiza* (Wang et al., 2013), the pathways for phenolic acid biosynthesis and relevant TFs are now receiving more research attention (Song and Wang, 2011; Zhang et al., 2013, 2014; Wang et al., 2015; Wang Z.J. et al., 2016; Xu et al., 2016).

Jasmonates (JAs) are integral to various defense responses that lead to the accumulation of secondary metabolites (Browse, 2009). MYC2 is a basic helix-loop-helix (bHLH) TF in the JA signaling pathway. Under stress-free growth conditions, jasmonate ZIM-domain (JAZ) proteins inhibit the function of TFs which are response to JA (Pauwels et al., 2010; Shyu et al., 2012). The JAZ proteins combine with TOPLESS (TPL) and TPL-related proteins to form a repressor complex, through direct or indirect interaction with the adaptor protein Novel Interactor of JAZ (NINJA) (Iván et al., 2013). In response to biotic or abiotic stresses, jasmonoyl-L-isoleucine (JA-Ile) is rapidly synthesized in plant tissues (Wasternack and Kombrink, 2010). The binding of JA-Ile to Coronatine Insensitive 1 (COI1)-JAZ co-receptor complexes results in the removal of JAZ repressors by the 26S proteasome (Sheard et al., 2010; Zhang et al., 2016). Destruction of JAZ repressors liberates MYC2 from suppression. The latter orchestrates the expression of many JA-responsive genes, including several TFs that have an important role in the JA signaling pathway (Kemal and Manners, 2013).

The roles of JA in controlling genes participated in the synthesis of secondary metabolites are well-established. MYC2 positively or negatively regulates secondary metabolism during JA signaling in a species-specific manner. For example, it can positively regulate the biosynthesis of flavonoid (e.g., anthocyanin) but inhibit JA-responsive biosynthesis of Trp-derived indole-glucosinolates in *Arabidopsis* plants with JA treatment (Dombrecht et al., 2007; Kemal and Manners, 2013). The MYC2 orthologs from tobacco species function as positive regulators of JA activation during nicotine biosynthesis in *Nicotiana benthamiana* (Todd et al., 2010; Kathleen et al., 2011). CrMYC2 regulates the expression of *ORCA* genes which are response to JA and the genes have an effect on alkaloid biosynthesis in *Catharanthus roseus* (Zhang et al., 2011). Results from ectopic RNA interference-mediated knockdown experiments with hairy root cultures of *S. miltiorrhiza* have suggested that SmMYC2a and SmMYC2b two MYC2 orthologs from that species promote the biosynthesis of tanshinones and Sal B (Zhou Y. et al., 2016). We previously isolated *SmMYC2* (GenBank accession number KJ945636) from *S. miltiorrhiza*. This gene encodes a putative MYC2, and its expression can be induced by methyl jasmonate (MeJA), light, or wounding (Zhou W.P. et al., 2016). However, it has not been clarified the effect of *SmMYC2* overexpression on *S. miltiorrhiza*.

Here, we investigated whether such overexpression might increase plant levels of tanshinones and Sal B. Our goal was to gain a better understanding of the role of MYC2 in *S. miltiorrhiza*, a valuable medicinal plant. The development of new strategies for genetic manipulation of these plants might be used to enhance the production of bioactive compounds. We conducted genome-wide transcriptional profiling of *SmMYC2-*overexpression transgenic *S. miltiorrhiza* and empty vector-transformed control plants, and also performed functional analyses. Roles were identified for MYC2 in the regulation of phenolic acids and tanshinones in that species. Moreover, overexpression of *SmMYC2* greatly improved the production of hydrophilic phenolic acids by activating not only the phenylpropanoid biosynthesis pathway but also the pathways for phenylalanine and tyrosine biosynthesis.

## Materials and Methods

### Experimental Materials

Seeds of *S. miltiorrhiza* were surface-sterilized as we have described previously (Yan and Wang, 2007). Sterile plants were cultured on an MS basal medium under the conditions presented earlier (Hua et al., 2011). All chemicals were purchased from Sigma Chemical Co. (St. Louis, MO, United States). Solvents were high-performance liquid chromatography (HPLC) grade. The standards (RA, Sal B, tanshinone IIA, and cryptotanshinone) used for HPLC were bought from the National Institute for the Control of Pharmaceutical and Biological Products (Beijing, China). We prepared the standards as stock solutions with methanol and put them at -18°C in a dark environment. The primers are listed in Supplementary Table 1.

### Vector Construction and Transformation

To construct *SmMYC2-*overexpression vectors, we used the pMD19T–*SmMYC2* construct already developed in our laboratory (Zhou W.P. et al., 2016). *SmMYC2* was amplified from pMD19T–*SmMYC2* with primers 207-*SmMYC2*-F/207-*SmMYC2*-R, which contain *att*B1/*att*B2 sites. Polymerase chain reaction (PCR) products were cloned into entry vector pDONR207, using the BP recombination reaction, and then transferred into destination vector pEarleyGate201 (Earley et al., 2006) through an LR reaction, according to the protocol from the Gateway technology manufacturer (Invitrogen, United States). The resulting pDONR207–*SmMYC2* and pEarleyGate201–*SmMYC2* were sequenced by Beijing Ao Ke Wei Ye Technologies Co., Ltd. (China).

The pEarleyGate201–*SmMYC2* construct was mobilized into *Agrobacterium tumefaciens* GV3101. Resistant colonies were verified by PCR-amplification and used for transformation experiments (Zhang et al., 2010). *Agrobacterium*-mediated gene transfer was performed according to the protocol established in our laboratory (Yan and Wang, 2007). The pEarleyGate 201 vector was also transformed by the same method used for control plants (CK) that were transformed with an empty vector.

### Transformant Selection and Molecular Characterization

After transformation, explants co-cultured with *A. tumefaciens* were transferred to an MS selection medium containing 1 mg L^-1^ naphthalene acetic acid, 10 mg L^-1^ 6-benzyl-aminopurine, 10 mg L^-1^ glufosinate-ammonium, and 200 mg L^-1^ cefotaxime. They were transferred to fresh selection media at 10-day intervals. As described previously, developing shoots were excised and placed on a 1/2-MS selection medium supplemented with 10 mg L^-1^ glufosinate-ammonium and 200 mg L^-1^ cefotaxime for root induction (Wang et al., 2013). A well-developed root system usually formed within 2 weeks. The rooted plants were cut into intermodal segments and propagated on a 1/2-MS basal medium (Wei et al., 2017).

Molecular characterization of transgenic plants was performed as described before (Wang H.Q. et al., 2016). Transgenic lines of the T1 generation were used for molecular analysis. Genomic DNA was extracted from the leaves of 1-month-old herbicide-resistant plants. Positive pEarleyGate201 transgenic lines and pEarleyGate201–*SmMYC2* transgenic lines were detected using 35S-F/R (Supplementary Table 1). All PCR reactions were performed as follows: preheating at 94°C for 3 min; then 32 cycles at 94°C for 30 s, 58°C for 40 s, and 72°C for 1 min; with a final extension at 72°C for 10 min. The target fragments for all transgenic lines were 721 bp long. The pEarleyGate201–*SmMYC2* vector served as the positive control, while genomic DNA from wild-type plants was the negative control.

Total RNA was isolated with a Plant RNA Kit (OMEGA, Houston, TX, United States), according to the manufacturer’s protocol, and was converted into cDNA using a primeScript^®^ RT Reagent Kit (TaKaRa, Japan). The reverse-transcription (RT) products were amplified with *SmMYC2*-RTF/R primers so that we could monitor gene expression via real-time quantitative PCR. A housekeeping gene, *SmUbiquitin*, served as the control and was amplified with Primers *SmUbiquitin*-F/R. Quantitative real-time reactions were performed in triplicate under the following conditions: one cycle of pre-denaturation at 95°C for 3 min, then 45 cycles of amplification at 95°C for 10 s and 60°C for 30 s. Relative expression for each gene was calculated by the comparative C_T_ method (Vandesompele et al., 2002).

### HPLC Analysis of Phenolic Compounds and Tanshinones

Roots were collected from 2-month-old plants and air-dried at 20 ± 2°C. We used standards of RA, Sal B, tanshinone IIA, and cryptotanshinone in HPLC experiments. Phenolic compounds and tanshinones were extracted as described before (Zhang et al., 2010). Extracts were analyzed with a C18 column (250 × 4.6 mm, 5 μm particle size) on an Agilent 1260 Infinity LC System (United States). The mobile phase comprised 0.4% acetic acid in water (A), acetonitrile (B), and methanol (C). The solvent gradient was as follows: 0–5 min: A 95–90% and B 5–10%; 5–25 min: A 90–67%, B 10–30%, and C 0–3%; 25–40 min: A 67–60%, B 30–5%, and C 3–5%; 40–60 min: A 60–35%, B 35–55%, and C 5–10%; 60–70 min: A 35–25%, B 55–60%, and C 10–15%; 70–75 min: A 25–10%, B 60–70%, and C 15–20%; and 75–80 min: A 10–0%, B 70–80%, and C 20–20%. The flow rate was adjusted to 1.0 mL min^-1^ and the detection wavelength was 280 nm. All separations were performed at 30°C.

### Determination of Total Phenolic and Total Flavonoid Concentrations

The amounts of total phenolics and flavonoids were determined from 2-month-old plants, as we have described previously (Zhang and Wang, 2009). Total phenolics were measured using a modified colorimetric Folin–Ciocalteu method and the absorbance was read at 765 nm. Calculations were based on a calibration curve obtained with gallic acid. The calibration equation for gallic acid was *y* = 0.0107*x* + 0.0752 (*R*^2^ = 0.9997). Total flavonoid content was determined by using a colorimetric method and the absorbance was measured at 510 nm. Calculations were based on a calibration curve obtained with (-)-epicatechin. The calibration equation for epicatechin was *y* = 1.113*x* + 0.0517 (*R*^2^ = 0.9971).

### Calculation of Jasmonate Concentration

The levels of JA were investigated by culturing samples of fresh leaves from transgenic line OEM-12 and CK plants. After the tissues were ground in liquid nitrogen, 9 mL of PBS buffer (pH 7.4) was added. The extracts were then centrifuged (8000 × *g* for 30 min at 4°C), and the upper layers were retained for our extractions, which were measured in triplicate. Endogenous JAs were measured in both transgenic and control plants using a Plant Jasmonic acid (JA) ELISA Kit (mlbio, China) according to the manufacturer’s protocol. A set of calibration standards was assayed to produce a standard curve of optical density (OD) versus JA concentration at 0, 125, 250, 500, 1000, and 2000 pmol L^-1^. The amount of JA in each sample was determined by comparing OD values with the standard curve. The intensity of the final reaction color was measured spectrophotometrically at 450 nm to calculate the final JA concentration.

### Transcriptome Analysis

Total RNA was isolated from 2-month-old control and transgenic plants (*n* = 3 each), using TRIzol^®^ Reagent (Invitrogen, United States) according to the manufacturer’s protocol. Samples from OEM-12 and CK plants were sequenced by Biomarker Technologies Co., Ltd. (Beijing, China). Three biological replicates were used for library preparation and RNAseq. Two samples were used for library construction and sequencing in triplicate. The cDNA library construction and Illumina HiSeq4000 sequencing were performed as described previously (Cao et al., 2016). Our raw data have been submitted to National Center for Biotechnology Information (NCBI) with the accession number of SRP113567.

### Identification of Differentially Expressed Genes (DEGs)

Total RNA-Seq reads were mapped to the *S. miltiorrhiza* genome. Expression levels of various genes were calculated by Cufflinks v2.2.1 (Trapnell et al., 2010); genes with value *Q* < 0.05, a false discovery rate (FDR) < 0.001, and an estimated absolute log_2_ fold-change (log_2_ FC) > 1.0 were selected. Blast X alignments were made (*E*-values < 0.00001) between the DEGs, using information from the protein databases NR, SwissProt, Pfam, and KEGG. For examining pathway enrichment, all DEGs were mapped to terms in the KEGG database to identify significantly over-represented metabolic pathways or signal transduction pathways as described before (Cao et al., 2016). We primarily focused on differentially regulated pathways closely related to the biosynthesis of salvianolic acids and tanshinones.

### Validation of RNA-seq Data by qRT-PCR

To validate the RNA-seq data, we selected 12 genes involved in salvianolic acid production, tanshinone biosynthesis, flavonoids biosynthesis, and the JA signaling pathway for our qRT-PCR experiments. *SmUbiquitin* served as the reference gene. Quantitative reactions were performed on a LightCycler^®^ 96 real-time PCR detection system (Roche, Switzerland), using SYBR Premix Ex TaqTM (TaKaRa). Relative expression for each gene was calculated by the comparative C_T_ method (Vandesompele et al., 2002). All assays for each gene were performed simultaneously, and in triplicate, under identical conditions.

### Statistical Analysis

The statistical evaluation was performed using SPSS version 17.0 software. All data were presented as the means ± standard error (SE) of at least three replicates. Analysis of variance (ANOVA) was followed by Tukey’s pairwise comparison tests. Mean values were considered significantly different at *P* < 0.05.

## Results

### Generation of Transgenic *Salvia miltiorrhiza* Plants

**FIGURE 1:**
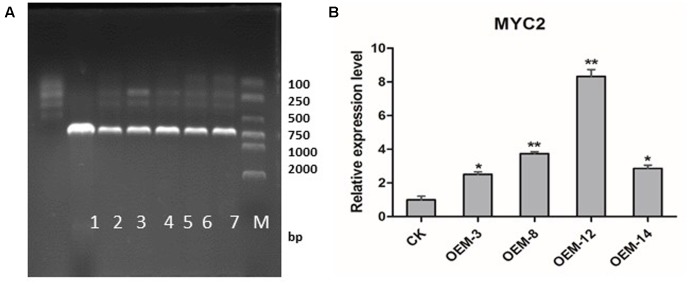
Expression of *SmMYC2* in control (CK) and overexpression transgenic lines. **(A)** PCR detection. M, DL2000 DNA marker; band sizes (bp) are shown on right side. Lanes 1, negative control; 2, positive control; 3–7, different transgenic lines. **(B)** Real-time quantitative PCR analysis; all data are means of three replicates, with error bars indicating SD; ^∗^ and ^∗∗^, values are significantly different from CK at *P* < 0.05 and *P* < 0.01, respectively.

Using *Agrobacterium*-mediated transformation protocol established in our laboratory (Yan and Wang, 2007), we generated transgenic *S. miltiorrhiza* plants that over-expressed *SmMYC2*. After selective culturing on an herbicide medium, those plants were confirmed through PCR amplification to contain an expected 721-bp fragment of the *CaMV 35S* promoter (**Figure 1A**). Real-time quantitative PCR demonstrated that *SmMYC2* was over-expressed in lines OEM-3, OEM-8, OEM-12, and OEM-14 (**Figure 1B**). Because expression was significantly higher in OEM-8 and OEM-12 than in the other lines and the CK (plants transformed with the empty vector), we chose them for further analysis.

### Overexpression of *SmMYC2* Enhances Production of Salvianolic and Rosmarinic Acids in Transgenic *S. miltiorrhiza*

**FIGURE 2:**
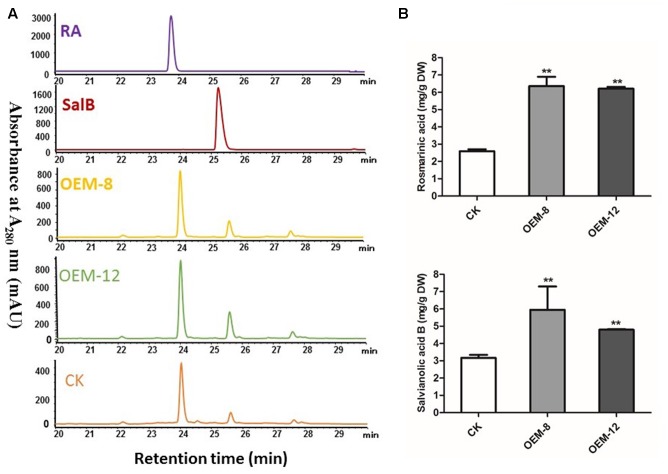
Overexpression of *SmMYC2* enhances production of rosmarinic acid (RA) and salvianolic acid B (Sal B) in *S. miltiorrhiza.* **(A)** Representative HPLCs of 2-month-old plants and standards at the wavelength of 280 nm: RA, standard of RA; Sal B, standard of Sal B; CK, control transformed with empty vector; OEM-8 and OEM-12, positive transgenic lines. **(B)** Concentrations of RA and Sal B in root extracts from CK and transgenic plants. All data are means of three replicates, with error bars indicating SD. ^∗∗^Values are significantly different from CK at *P* < 0.01.

To characterize how production of phenolic acids and tanshinones is modified in plants over-expressing *SmMYC2*, we extracted them and subjected them to HPLC separation. Overexpression tended to improve their biosynthesis. In the roots of 2-month-old plants, RA and its dimer Sal B were the major components, showing respective retention times of 24.02 ± 0.02 and 25.60 ± 0.02 min (**Figure 2A**). Compared with levels in CK samples, the concentrations of both RA and Sal B were increased significantly in the transgenics (*P* < 0.01) (**Figure 2B**), particularly in OEM-12, which had mean levels of 6.36 ± 0.21 mg g^-1^ RA and 5.95 ± 0.07 mg g^-1^ Sal B. RA and Sal B concentrations in CK roots were 2.59 ± 0.04 and 3.17 ± 0.01 mg g^-1^, respectively. Compared with CK, the OEM-12 showed a 2.46-fold increase in RA and a 1.88-fold increase in Sal B. In OEM-8, concentrations of RA and Sal B were 6.21 ± 0.03 and 4.80 ± 0.01 mg g^-1^, respectively, which was approximately 2.40- and 1.51-fold higher than those of the CK.

The two tanshinones investigated here are key active ingredients in danshen. Retention times were 75.2 ± 0.2 min for tanshinone IIA and 68.1 ± 0.3 min for cryptotanshinone. However, neither was detected by HPLC in the 2-month-old OEM lines and control plants (Supplementary Figure 1).

### Transgenic Plants Show Higher Levels of Total Phenolics and Total Flavonoids

**FIGURE 3:**
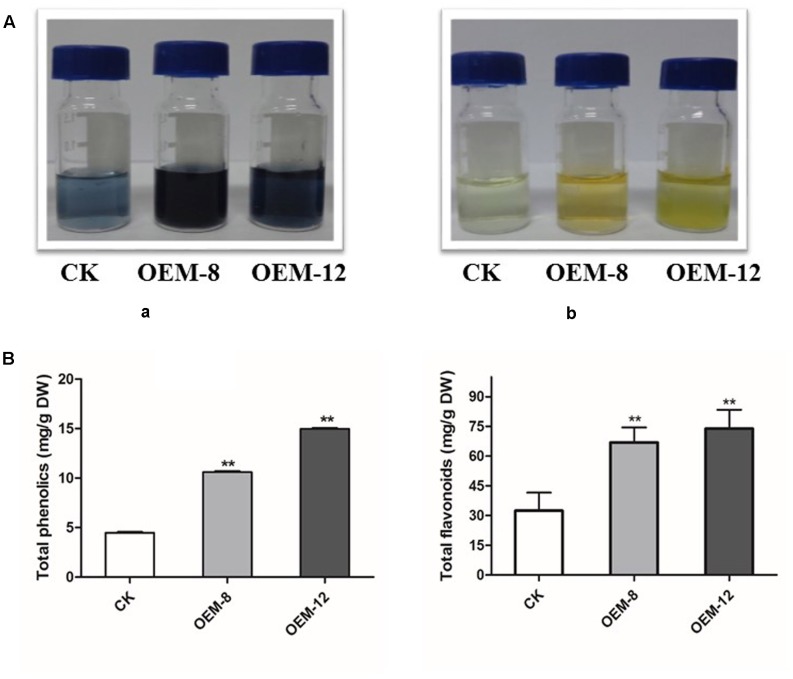
Concentrations of total phenolics and total flavonoids in transgenic lines and CK, control. **(A)** Visible signs of total phenolics **(a)** and total flavonoids **(b)**. **(B)** Total accumulations in root extracts. All data are means of three replicates, with error bars indicating SD. ^∗∗^Values are significantly different from CK at *P* < 0.01.

Our results presented above demonstrated that overexpression of *SmMYC2* modified the accumulation of two non-flavonoid phenolic acids, RA and Sal B. To evaluate whether this upregulation induced the phenylpropanoid pathway and provided substrates for the biosynthesis of other types of end products, we performed global assays for phenolics and flavonoids which share an upstream core phenylpropanoid metabolism with Sal B. As showed in **Figure 3A**, morphological differences were apparent among the tested lines. Both types of compounds accumulated at higher levels in the roots of the overexpression lines than in CK samples. Concentrations of total phenolics and total flavonoids were 4.47 ± 0.13 and 32.43 ± 10.31 mg g^-1^ for CK, 10.61 ± 0.21 and 66.84 ± 8.48 mg g^-1^ for OEM-8; and 14.98 ± 0.11 and 73.91 ± 12.31 mg g^-1^ for OEM-12, respectively (**Figure 3B**).

### Transcriptomic Analysis of *SmMYC2*-Overexpression and Control Plants of *S. miltiorrhiza*

For further investigation into the molecular mechanisms involved in the enhancement of salvianolic acid production in *SmMYC2*-overexpression transgenics, we compared the global expression profiles of OEM-12 versus CK. Two cDNA libraries were sequenced by Illumina deep sequencing to obtain approximately 31.26 and 25.93 million high-quality clean reads for OEM-12 and CK, respectively. Each read averaged 296 bp long. The Q30 value (percentage of sequences with a sequencing error rate < 0.1%) was 93.01% for OEM-12 and 92.86% for CK.

We analyzed the expression of unigenes using Bowtie (Langmead et al., 2009) and RSEM software (Li and Dewey, 2011) and normalized the values by Fragments Per Kilobase of transcript per Million mapped reads (FPKM) (Trapnell et al., 2010). Transcriptome analysis identified 2694 DEGs, with 1540 unigenes being up-regulated and 1154 down-regulated in OEM-12 when compared with expression in CK. The top 20 most up-regulated and most down-regulated genes are listed in Supplementary Tables 2 and 3.

**FIGURE 4:**
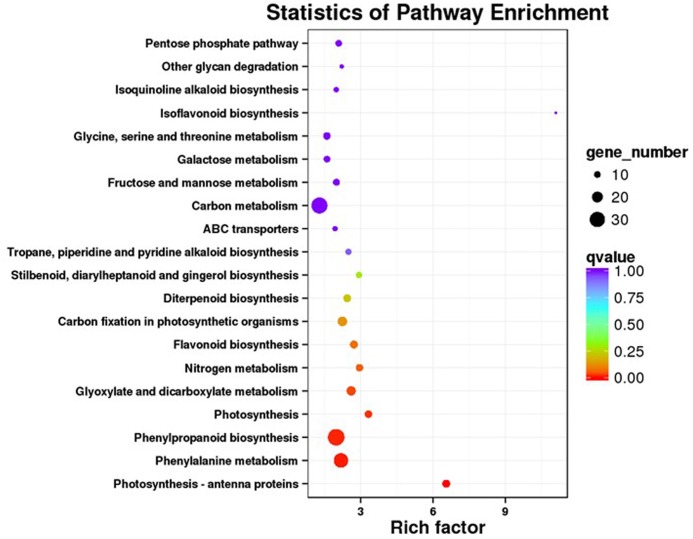
KEGG pathway enrichment analysis of DEGs.

Our KEGG analysis enabled us to identify 20 significantly enriched metabolic pathways for those DEGs (**Figure 4**). To examine the molecular basis for stimulation of phenolic acid production, we focused on biosynthetic pathways for phenolics, phenylalanine, tyrosine, and JA.

### DEGs Involved in JA Biosynthesis and Calculation of Endogenous JA Levels

**FIGURE 5:**
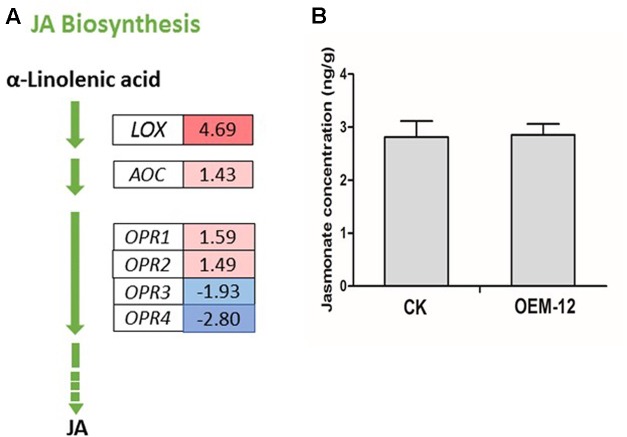
Changes in JA concentrations were not significant for transgenic plants over-expressing *SmMYC2*. **(A)** MYC2-mediated transcriptional activation of genes for JA metabolic enzymes. Blue boxes, putative encoding genes are down-regulated; red boxes, putative encoding genes are up-regulated. **(B)** Concentrations of JA in leaf extracts from CK control and transgenic line OEM-12.

Jasmonates play an integral role in various defense responses, leading to the accumulation of secondary metabolites (Browse, 2009). MYC2, a bHLH TF, acts as a transcriptional activator of JA-responsive genes that encode JA metabolic enzymes such as lipoxygenase (LOX), allene oxide synthase, allene oxide cyclase (AOC), 12-oxophytodienoate reductase (OPR), JA-amido synthetase (also known as JASMONATE RESISTANCE1), and cytochrome P450 monooxygenase 94B3 (CYP94B3) (Lorenzo et al., 2004; Sasaki-Sekimoto et al., 2013). We investigated changes in the expression of genes closely associated with α-linolenic acid metabolism, which finally leads to JA biosynthesis, and found six DEGs for the latter pathway (**Figure 5A**). Two DEGs encoding putative LOX and AOC showed increased transcript abundance. Among the DEGs encoding putative OPR, two were transcriptionally activated while two were transcriptionally suppressed. We then applied ELISA techniques to determine the concentrations of endogenous JA in fresh leaves from OEM-12 and CK plants. Based on the OD values of our samples, we learned that JA levels were not significantly changed by overexpression of *SmMYC2*, with values of 2.85 ± 0.23 ng g^-1^ for Line OEM-12 versus 2.81 ± 0.21 ng g^-1^ for CK (**Figure 5B**).

### DEGs Involved in Pathway for Salvianolic Acid Biosynthesis

**FIGURE 6:**
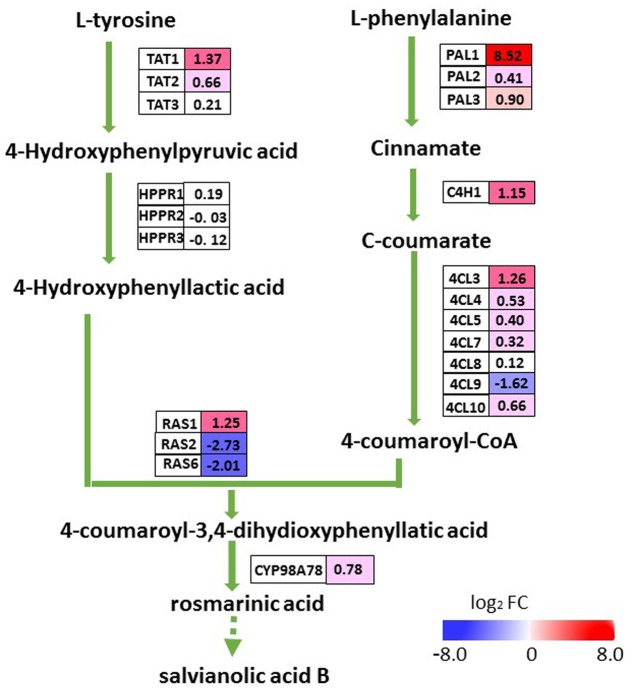
DEGs involved in pathway for salvianolic acid biosynthesis. For each gene, relative expression (OEM-12 versus control) is represented in log_2_ FC. Blue boxes, putative encoding genes are down-regulated; red boxes, putative encoding genes are up-regulated.

To evaluate whether the elevated accumulation of salvianolic acids in transgenic lines was a result of up-regulated expression of *SmMYC2*, we examined transcript levels for all of the putative enzyme genes in the pathway for salvianolic acid biosynthesis (**Figure 6**). The transcriptome database revealed 21 genes homologous to *SmPAL*, *SmC4H*, *Sm4CL*, *SmTAT*, *SmHPPR*, *SmRAS*, and *SmCYP98A78* (N.B., PAL, phenyl-alanine ammonia-lyase; C4H, cinnamate 4-hydroxylase; 4CL, 4-coumarate: coenzyme A ligase; TAT, tyrosine amino-transferase; HPPR, hydroxyphenylpyruvate reductase; and RA, rosmarinic acid synthase). Among the eight DEGs in the salvianolic acid biosynthesis pathway, five (*SmTAT1*, *SmPAL1*, *SmC4H1*, *Sm4CL3*, and *SmRAS1*) were up-regulated in the roots of OEM-12 while three (*Sm4CL9*, *SmRAS2*, and *SmRAS6*) were down-regulated. In that line, transcription was increased by 367.1-fold for *SmPAL1*, a critical gene for the synthesis of major water-soluble pharmaceutical ingredients within *S. miltiorrhiza* (Song and Wang, 2011). Expression for most of the other genes that were not differentially expressed slightly increased in OEM-12, according to the transcriptome data. Thus, upregulation of genes in the pathway for salvianolic acid biosynthesis was consistent with the elevated concentrations of salvianolic acids detected in the roots of transgenic plants.

### DEGs Involved in Phenylalanine and Tyrosine Biosynthesis

**FIGURE 7:**
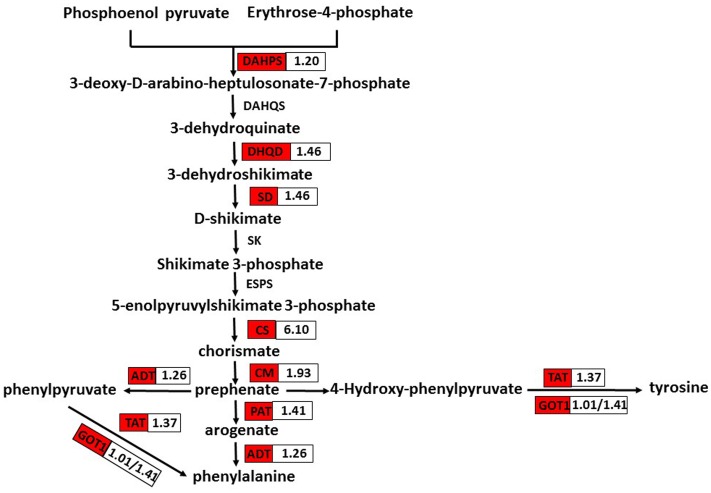
DEGs involved in phenylalanine and tyrosine biosynthesis. Red boxes, putative encoding genes are up-regulated. CS, chorismate synthase; CM, chorismate mutase; PAT, bifunctional aspartate aminotransferase and glutamate/aspartate-prephenate aminotransferase; ADT, arogenate/prephenate dehydratase; GOT1, aspartate aminotransferase; and TAT, tyrosine aminotransferase.

Phenolic secondary metabolites and their precursors are synthesized via the pathway of shikimate biosynthesis and its numerous branch points. That pathway is closely interlinked with those of the aromatic amino acids (L-tryptophan, L-phenylalanine, and L-tyrosine) (Tohge et al., 2013). Phenylalanine and tyrosine are precursors of RA and its derivative Sal B. In *S. miltiorrhiza*, the phenolic acid biosynthetic pathway starts from the general phenylpropanoid pathway and the tyrosine-derived pathway (Peng et al., 2013). Therefore, phenylalanine and tyrosine are of great importance. We found that nine DEGs related to their pathways were up-regulated in OEM-12 (**Figure 7**). Therefore, activation of the pathways for phenylalanine and tyrosine biosynthesis, which then provides more precursors for phenolic acids, may contribute to the improvement in RA and Sal B concentrations.

### DEGs Involved in Tanshinone Biosynthesis

**FIGURE 8:**
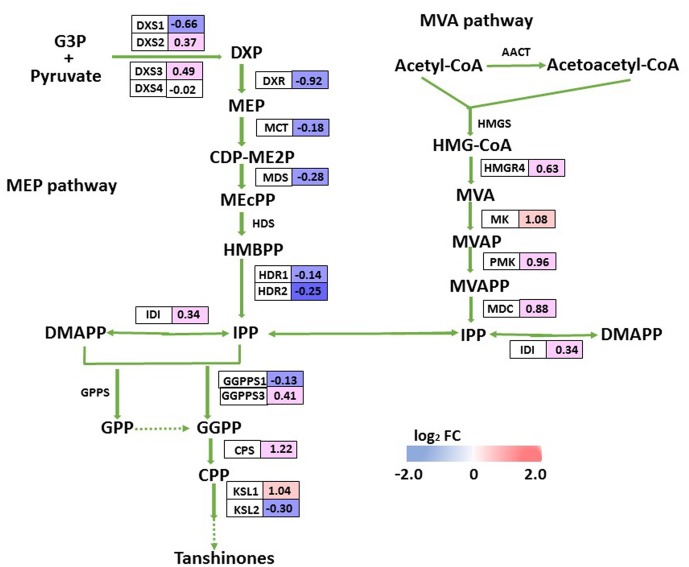
DEGs involved in tanshinone biosynthesis. For each gene, relative expression (OEM-12 versus control) is represented in log_2_ FC. Blue boxes, putative encoding genes are down-regulated; red boxes, putative encoding genes are up-regulated.

Tanshinones are derived from their precursor isopentenyl pyrophosphate (IPP), which is produced by two biosynthesis pathways: the mevalonate (MVA) pathway in the cytosol and the methylerythritol phosphate (MEP) pathway in the plastids. Although we did not detect tanshinone IIA or cryptotanshinone by HPLC in 2-month-old overexpression lines or control plants, we monitored the transcript levels of putative enzyme genes in the tanshinone biosynthesis pathway, and identified only three DEGs (*SmCPS*, *SmKSL1*, and *SmMK*) there, all of which were up-regulated in OEM-12. We found it interesting that expression for most MEP pathway genes was slightly decreased while most MVA pathway genes were somewhat induced (**Figure 8**). This implied that the pathway for tanshinone biosynthesis was not obviously activated in the transgenics.

### G-Box Analysis of Related Genes

MYC2 is both directly and indirectly involved in regulating secondary metabolites. The G-box fragment (5′-CACGTG-3′) is the preferred core binding site of MYC2, followed by the 5′-CACGTT-3′ (∼35% of binding activity of 5′-CACGTG-3′) and 5′-CACATG-3′ (∼20% of binding activity of 5′-CACGTG-3′) sequences (Shoji and Hashimoto, 2011; Kemal and Manners, 2013). We analyzed the promoter sequences of 29 putative genes for enzymes in the pathway for salvianolic acid biosynthesis and found that many, e.g., *SmTAT1*, *SmHPPR1*, *SmCYP98A78*, and *SmRAS2*, contain G-box sequences in their promoters (Supplementary Table 4). This suggests that MYC2 directly binds to the promoter of each of those genes to regulate their expression. Moreover, *SmPAL1* has only a G-box-like sequence (5′-CATCTG-3′) in its promoter, and its transcript levels increased 367.1-fold in our OEM-12 transgenic line. Additional studies, such as chromatin immunoprecipitation (ChIP) sequencing analyses, are needed for genome-wide identification of direct MYC2 targets in *S. miltiorrhiza* (Kemal and Manners, 2013).

### Confirmation of MYC2-Mediated Transcriptional Changes by Quantitative RT-PCR

**FIGURE 9:**
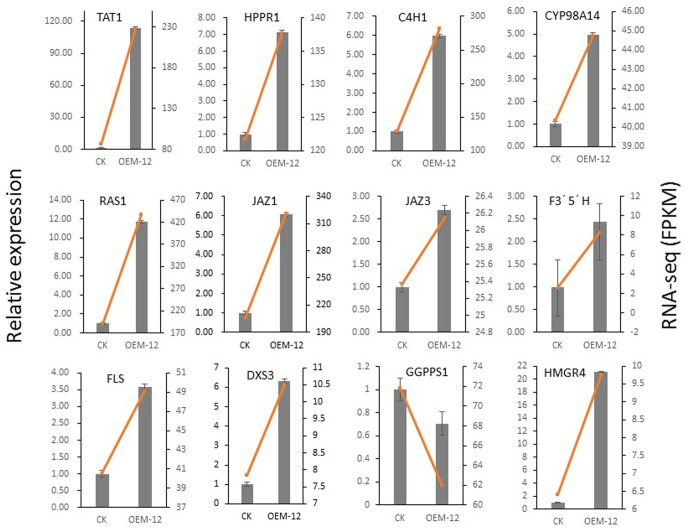
Validation by qRT-PCR of 12 genes involved in phenolic acid biosynthesis, JA signaling pathway, and tanshinone biosynthesis in control and transgenic line OEM-12.

To verify that these transcriptional changes were induced by MYC2, we examined 12 genes involved in phenolic acid biosynthesis (*TAT*, *PAL*, *HPPR*, *C4H*, and *RAS*), the JA signaling pathway (*JAZ1* and *JAZ3*), tanshinone biosynthesis (*DXS3*, *GGPPS1*, and *HMGR4*), and flavonoids biosynthesis (*FLS* and *F3*′*5*′*H*). Their expression patterns, as detected by qRT-PCR, were consistent with those obtained from the RNA-Seq data (**Figure 9**). Overall, this qRT-PCR analysis confirmed that the RNA-Seq results were statistically reliable and comparable to transcriptomic data.

## Discussion

Jasmonates act as conserved elicitors of plant secondary metabolism. Exogenous application of MeJA triggers an extensive transcriptional reprogramming of metabolism and dramatically increases the biosynthesis of active ingredients in *S. miltiorrhiza* (Ge et al., 2015). Accordingly, MYC2 serves as a regulatory hub within the JA signaling pathway and regulates secondary metabolism (Kemal and Manners, 2013). *At*MYC2 in *Arabidopsis thaliana* modulates the biosynthesis of glucosinolate, insect activity, and feeding behavior (Schweizer et al., 2013). In *Nicotiana tabacum*, *Nt*MYC2a and *Nt*MYC2b control multiple JA-inducible steps in nicotine biosynthesis (Zhang et al., 2012; Zhou Y. et al., 2016). Furthermore, *Md*MYC2 positively regulates flavonoid biosynthesis by modulating the expression of positive regulators in *Malus domestica* during JA signaling (An et al., 2016). Here, we investigated the function of SmMYC2 in regulating the production of active ingredients by over-expressing *SmMYC2* in *S. miltiorrhiza*, followed by metabolic analysis and RNA-seq.

Positive regulation by *Sm*MYC2 of phenolic acid biosynthesis and overexpression of *SmMYC2* significantly improved the concentrations of RA and Sal B calculated in *S. miltiorrhiza*. Transcriptome analysis showed that such overexpression activated not only the phenolic acid biosynthetic pathway but also the pathways for phenylalanine and tyrosine biosynthesis. Both *Sm*MYC2a and *Sm*MYC2b are positive regulators of multiple genes in the pathways for tanshinone and phenolic acid biosynthesis in *S. miltiorrhiz* (Zhou Y. et al., 2016). Because we did not detect either tanshinone IIA or cryptotanshinone in any of our tested genotypes, this seemed to indicate that the tanshinone biosynthetic pathway is not obviously activated in OEM-12, based on the results of RNA-seq. Transcript levels for *SmMYC2* were 3.77-fold higher in OEM-8 and 8.39-fold higher in OEM-12 when compared with CK. However, that enhancement was not as high as we had expected, possibly because MYC2 negatively regulates its own expression (Dombrecht et al., 2007). Therefore, we might speculate that *Sm*MYC2 prioritizes the phenolic acid biosynthetic pathway over the tanshinone biosynthetic pathway.

Rosmarinic acid is an ester of 3,4-dihydroxyphenyllactic acid and caffeic acid. Those two compounds are synthesized via the tyrosine-derived pathway and the phenylpropanoid pathway, respectively (Petersen et al., 1993; Song and Wang, 2011). The latter pathway includes PAL, C4H, and 4CL, while the former includes TAT and HPPR. Line OEM-12 showed elevated accumulations of RA and SalB, demonstrating the upregulation of most enzyme genes in the pathway for phenolic acid biosynthesis. The first enzyme in that pathway is PAL, and PAL1 plays a major role during the development and metabolic course of *S. miltiorrhiza* (Song and Wang, 2011). The transcription level of *SmPAL1* increased by 367.1-fold in OEM-12 according to the transcriptome data. *SmTAT1* was previously identified as being involved in RA biosynthesis in hairy root cultures of *S. miltiorrhiza* (Xiao et al., 2011). Our qRT-PCR results showed that the transcript level of *SmTAT1* was 110-fold higher in OEM-12 than in CK. This strong induction of *SmPAL1* and *SmTAT1* contributed to the accumulation of phenolic acids. The coupling of products from the phenylpropanoid and tyrosine-derived pathways is catalyzed by RAS, and we identified six *SmRAS*s in the *S. miltiorrhiza* genome. Based on TBLASTN with amino acid sequences from other plant species, only *SmRAS1* has been verified to form the precursor of RA in *S. miltiorrhiza* (Peng et al., 2013; Wang et al., 2015). We noted here that transcripts of *SmRAS1* increased while those of *SmRAS2* and *SmRAS6* decreased in OEM-12. Although the *S. miltiorrhiza* genome contains at least 29 genes related to phenolic acid biosynthesis (Wang et al., 2015), most of them, including *SmRAS2* and *SmRAS6*, have not yet been experimentally proven to participate in that process.

It is well known that exogenous JAs can up-regulate the expression of MYC2 and constitutive expression of *AtMYC2* in the wild-type background induced an exaggerated response to JA (Lorenzo et al., 2004), but we don’t know if overexpression of MYC2 will influence the endogenous JA biosynthesis. We compared the JA levels between CK and OEM-12. The results showed that overexpression of *SmMYC2* did not significantly change the content of endogenous JA. Besides, our transcriptome analysis showed that among the six DEGs in JA biosynthesis pathway, four (*LOX*, *AOC*, *OPR1*, and *OPR2*) were up-regulated in OEM-12 while two (*OPR3* and *OPR4*) were down-regulated. The transcriptome data suggested that the pathway for JA biosynthesis was vibrated, but not obviously activated in OEM-12, which was consistent with the JA levels.

It was verified that two MYC2 orthologs from *S. miltiorrhiza* positively regulate the biosynthesis of tanshinones and Sal B by ectopic RNA interference mediated knockdown experiments with hairy root cultures (Zhou Y. et al., 2016), but it did not mean that overexpression of *SmMYC2* would certainly increase the contents of tanshinones and phenolic acids in transgenic plantlets. Our results showed that overexpression of *SmMYC2* significantly increased the contents of phenolic acids by activating not only the phenylpropanoid biosynthesis pathway but also the pathways for phenylalanine and tyrosine biosynthesis. Overall, although the molecular mechanism by which MYC2 regulates phenolic acid biosynthesis in *S. miltiorrhiza* is still unknown, our research results indicate that it has an important role in modulating this biosynthesis. It actives pathways for both secondary and primary metabolism. Therefore, our study of *SmMYC2* overexpression in *S. miltiorrhiza* provides a good foundation for elucidating the molecular mechanism regulated by MYC2.

## Author Contributions

The experiments were conceived and organized by XC and ZW. NY and WZ performed the experiments. JS, XW, and LL contributed to the data analysis. The paper was written by NY, LW, and XC. All authors discussed and approved the final manuscript.

## Conflict of Interest Statement

The authors declare that the research was conducted in the absence of any commercial or financial relationships that could be construed as a potential conflict of interest.
