# Supplementary material for: Overexpression of SmMYC2 Increases the Production of Phenolic Acids in Salvia miltiorrhiza
Source: Front Plant Sci. 2017 Oct 18;8:1804. doi: 10.3389/fpls.2017.01804 (PMC5708653; doi:10.3389/fpls.2017.01804)
Supplement: Supplementary file 1 [file DataSheet_1.DOCX]

***Supplementary material***

**Overexpression of *SmMYC2* increases the production of phenolic acids in *Salvia miltiorrhiza***

**Na Yang^*^, Wenping Zhou, Jiao Su, Xiaofan Wang, Lin Li, Liru Wang**

*** Correspondence:** Corresponding Author:

Xiaoyan Cao: [caoxiaoyan@snnu.edu.cn](mailto:caoxiaoyan@snnu.edu.cn).

Zhezhi Wang: [zzwang@snnu.edu.cn](mailto:zzwang@snnu.edu.cn).


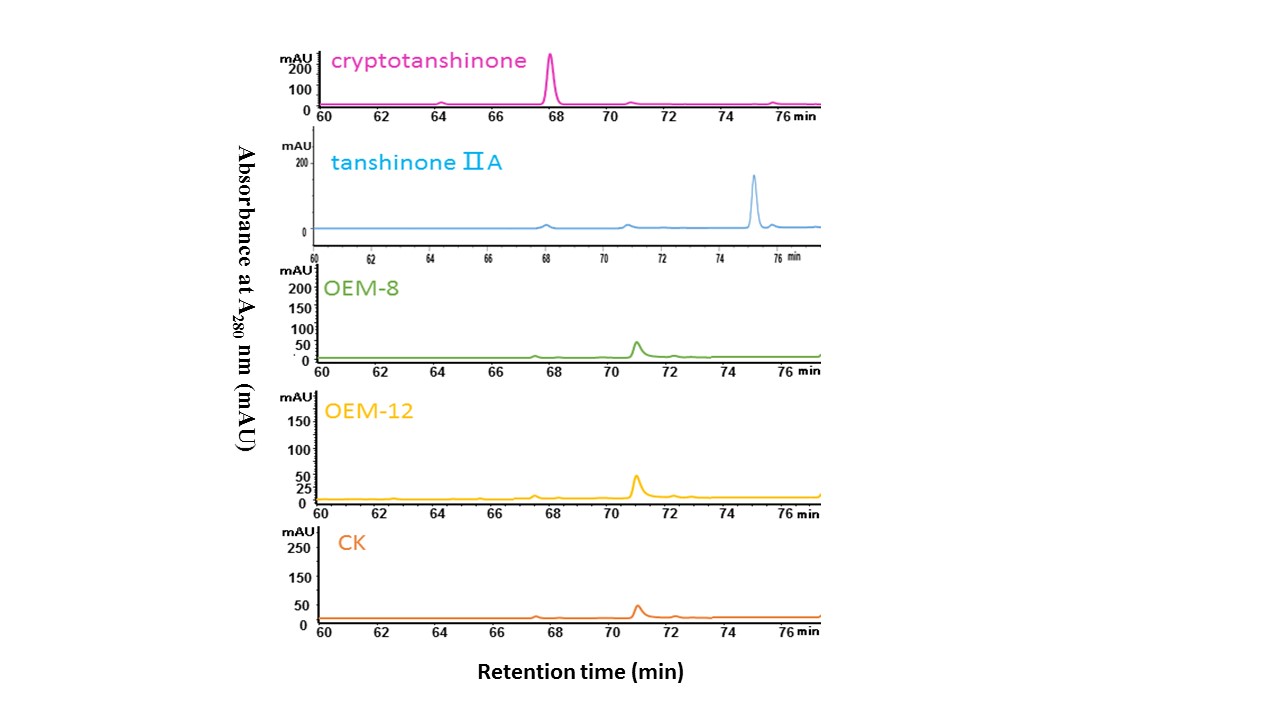


**Supplementary Figure 1** Representative HPLC chromatograms of 2-month-old *S.miltiorrhiza lines*. CK: transgenic plants transformed with empty vector; OEM-8 and OEM-12: positive transgenic lines overexpressing *SmMYC2*.
