# Supplementary material for: Overexpression of SmMYC2 Increases the Production of Phenolic Acids in Salvia miltiorrhiza
Source: Front Plant Sci. 2017 Oct 18;8:1804. doi: 10.3389/fpls.2017.01804 (PMC5708653; doi:10.3389/fpls.2017.01804)
Supplement: Supplementary file 2 [file DataSheet_2.DOCX]

***Supplementary material***

**Overexpression of *SmMYC2* increases the production of phenolic acids in *Salvia miltiorrhiza***

**Na Yang^*^, Wenping Zhou, Jiao Su, Xiaofan Wang, Lin Li, Liru Wang**

*** Correspondence:** Corresponding Author:

Xiaoyan Cao: [caoxiaoyan@snnu.edu.cn](mailto:caoxiaoyan@snnu.edu.cn).

Zhezhi Wang: [zzwang@snnu.edu.cn](mailto:zzwang@snnu.edu.cn).

**Supplementary Table 1** Primer pairs used in the paper

| Primer name | Sequence (5'→3') | Note |
| --- | --- | --- |
| *SmMYC2*-F | ATGGAATGATTGATTACCGCAC | Molecular cloning(*SmMYC2*) |
| *SmMYC2*-R | GGAGGAAACTAGACACAAAATACACC |  |
| 207-*SmMYC2*-F | GGGGACAAGTTTGTACAAAAAAGCAGGCTTCATGATTGATTACCGCACGCC | Vector Construction |
| 207-*SmMYC2*-R | GGGGACCACTTTGTACAAGAAAGCTGGGTCTCTAATCTCAGCAACTTTAGATATCA |  |
| *JCMYC2*-F | TACAAAGGCGGCAACAAACG | Transformant selection |
| *JCMYC2*-R | GCAATGGAATCCGAGGAGGT |  |
| Sm*4CL1*-RTF | TCGCCAAATACGACCTTTCC | qRT-PCR(Sm*4CL1*) |
| Sm*4CL1*-RTR | TGCTTCAGTCATCCCATACCC |  |
| *SmC4H*-RTF | CCAGGAGTCCAAATAACAGAGCCG | qRT-PCR(*SmC4H*) |
| *SmC4H*-RTR | GCCACCAAGCGTTCACCAAGAT |  |
| *SmCYP98A14*-RTF | ACGTGCGTGTTGCTACGAGAC | qRT-PCR(*SmCYP98A14*) |
| *SmCYP98A14*-RTR | CGTCGCCAGTGCTGCAACTAA |  |
| *SmF3'* *5'* *H*-RTF | CATCTACTCCAACATCGGACAGC | qRT-PCR(*SmF3'* *5'* *H*) |
| *SmF3'* *5'* *H*-RTR | CCCCACATAAGGTTCATCAACAG |  |
| *SmFLS*-RTF | GTTCGTGCATCCCGAGTTCAA | qRT-PCR(*SmFLS*) |
| *SmFLS*-RTR | CTTCTCGTCACCTCTCGACATCTTT |  |
| *SmHPPR*-RTF | TGACTCCAGAAACAACCCACATT | qRT-PCR(*SmHPPR*) |
| *SmHPPR*-RTR | CCCAGACGACCCTCCACAAG |  |
| *SmTAT*-RTF | CAACTGCTGGTCTTCCACAAAC | qRT-PCR(*SmTAT*) |
| *SmTAT*-RTR | GCGAGCCAAAACGGACA |  |
| *SmJAZ1*-RTF | GAGTGAGCCGAAACCAGAAAA | qRT-PCR(*SmJAZ1*) |
| *SmJAZ1*-RTR | GGAAGGATAGCTGGTGGCTAAA |  |
| *SmJAZ3*-RTF | ACGGTGACAACATACGGAGACA | qRT-PCR(*SmJAZ3*) |
| *SmJAZ3*-RTR | TCGCCTTAGGAGCCACTGAAT |  |
