# Supplementary material for: Overexpression of SmMYC2 Increases the Production of Phenolic Acids in Salvia miltiorrhiza
Source: Front Plant Sci. 2017 Oct 18;8:1804. doi: 10.3389/fpls.2017.01804 (PMC5708653; doi:10.3389/fpls.2017.01804)
Supplement: Supplementary file 3 [file DataSheet_3.DOCX]

***Supplementary material***

**Overexpression of *SmMYC2* increases the production of phenolic acids in *Salvia miltiorrhiza***

**Na Yang^*^, Wenping Zhou, Jiao Su, Xiaofan Wang, Lin Li, Liru Wang**

*** Correspondence:** Corresponding Author:

Xiaoyan Cao: [caoxiaoyan@snnu.edu.cn](mailto:caoxiaoyan@snnu.edu.cn).

Zhezhi Wang: [zzwang@snnu.edu.cn](mailto:zzwang@snnu.edu.cn).

**Supplementary Table 2** List of top 20 up-regulated genes in the transcriptome of transgenic *S. miltiorrhiza* line OEM-12

| Gene ID | log2FC | Annotation |
| --- | --- | --- |
| SMil_00014735 | 9.708879 | unnamed protein product [Coffea canephora] |
| Salvia_newGene_4301 | 9.382015 | uncharacterized protein LOC101253719 isoform X3 [Solanum lycopersicum] |
| SMil_00016860 | 8.953675 | uncharacterized protein LOC101262187 [Solanum lycopersicum] |
| SMil_00003437 | 8.879448 | B3 domain-containing transcription factor ABI3 isoform X1 |
| SMil_00024729 | 8.726794 | uncharacterized protein LOC105177468 [Sesamum indicum] |
| SMil_00013941 | 8.588958 | PREDICTED: 60S ribosomal protein L28-1-like [Sesamum indicum] |
| SMil_00019884 | 8.519342 | phenylalanine ammonia-lyase |
| Salvia_newGene_5383 | 8.36555 | WRKY transcription factor 40 |
| SMil_00011502 | 8.349551 | hypothetical protein MIMGU_mgv1a001107mg [Erythranthe guttata] |
| SMil_00018008 | 8.287084 | hypothetical protein MIMGU_mgv1a017627mg [Erythranthe guttata] |
| SMil_00003216 | 8.077722 | hypothetical protein MIMGU_mgv1a010701mg [Erythranthe guttata] |
| Salvia_newGene_238 | 7.96463 | myb-binding protein 1A-like protein |
| Salvia_newGene_2517 | 7.943468 | hypothetical protein CICLE_v10032869mg |
| SMil_00013561 | 7.917657 | thionin |
| Salvia_newGene_2245 | 7.904165 | vegetative cell wall protein gp1-like |
| SMil_00026998 | 7.846481 | putative late blight resistance protein homolog R1B-16 |
| SMil_00003061 | 7.776405 | SMLII |
| Salvia_newGene_5884 | 7.757128 | MYB-related transcription factor |
| Salvia_newGene_1355 | 7.747393 | hypothetical protein VITISV_021887 [Vitis vinifera] |
| Salvia_newGene_4198 | 7.747393 | PREDICTED: uncharacterized protein LOC103940543 |
