# Supplementary material for: Overexpression of SmMYC2 Increases the Production of Phenolic Acids in Salvia miltiorrhiza
Source: Front Plant Sci. 2017 Oct 18;8:1804. doi: 10.3389/fpls.2017.01804 (PMC5708653; doi:10.3389/fpls.2017.01804)
Supplement: Supplementary file 4 [file DataSheet_4.DOCX]

***Supplementary material***

**Overexpression of *SmMYC2* increases the production of phenolic acids in *Salvia miltiorrhiza***

**Na Yang^*^, Wenping Zhou, Jiao Su, Xiaofan Wang, Lin Li, Liru Wang**

*** Correspondence:** Corresponding Author:

Xiaoyan Cao: [caoxiaoyan@snnu.edu.cn](mailto:caoxiaoyan@snnu.edu.cn).

Zhezhi Wang: [zzwang@snnu.edu.cn](mailto:zzwang@snnu.edu.cn).

**Supplementary Table 3** List of top 20 down-regulated genes in the transcriptome of transgenic *S. miltiorrhiza* line OEM12

| Gene ID | log2FC | Annotation |
| --- | --- | --- |
| Salvia_newGene_3851 | -8.70996 | uncharacterized protein LOC103438319 [Malus domestica] |
| SMil_00027350 | -8.66427 | unnamed protein product |
| SMil_00016144 | -8.27661 | hypothetical protein MIMGU_mgv1a019789mg, partial [Erythranthe guttata] |
| SMil_00010669 | -8.0323 | probable ATP-dependent RNA helicase DDX11 isoform X2 [Sesamum indicum] |
| SMil_00017999 | -7.70463 | hypothetical protein MIMGU_mgv1a020957mg, partial [Erythranthe guttata] |
| Salvia_newGene_5712 | -7.49167 | hypothetical protein [Beta vulgaris subsp. vulgaris] |
| SMil_00012382 | -7.27679 | myeloid leukemia factor 1 [Sesamum indicum] |
| SMil_00019148 | -7.20261 | pto-interacting protein 1-like [Setaria italica] |
| SMil_00027246 | -7.17121 | BRASSINOSTEROID INSENSITIVE 1-associated receptor kinase 1-like |
| Salvia_newGene_4957 | -7.10443 | uncharacterized protein LOC105161706 isoform X2 |
| SMil_00029027 | -7.04866 | hypothetical protein MIMGU_mgv1a017822mg [Erythranthe guttata] |
| SMil_00017786 | -6.68604 | laccase-14-like [Sesamum indicum] |
| SMil_00017583 | -6.48313 | UDP-glycosyltransferase 74E2-like |
| SMil_00022419 | -5.8008 | uncharacterized protein LOC105173069 [Sesamum indicum] |
| SMil_00021186 | -5.79788 | 1-aminocyclopropane-1-carboxylate oxidase 5-like |
| SMil_00008878 | -5.71594 | hypothetical protein MIMGU_mgv1a026927mg [Erythranthe guttata] |
| SMil_00027118 | -5.69998 | unnamed protein product [Vitis vinifera] |
| SMil_00007448 | -5.64082 | hypothetical protein JCGZ_00233 [Jatropha curcas] |
| SMil_00006710 | -5.58682 | O-glucosyltransferase rumi homolog isoform X1 [Sesamum indicum] |
| SMil_00018989 | -5.57727 | CCR4-associated factor 1 homolog 7 [Sesamum indicum] |
