# Supplementary material for: Overexpression of SmMYC2 Increases the Production of Phenolic Acids in Salvia miltiorrhiza
Source: Front Plant Sci. 2017 Oct 18;8:1804. doi: 10.3389/fpls.2017.01804 (PMC5708653; doi:10.3389/fpls.2017.01804)
Supplement: Supplementary file 5 [file DataSheet_5.DOCX]

***Supplementary material***

**Overexpression of *SmMYC2* increases the production of phenolic acids in *Salvia miltiorrhiza***

**Na Yang^*^, Wenping Zhou, Jiao Su, Xiaofan Wang, Lin Li, Liru Wang**

*** Correspondence:** Corresponding Author:

Xiaoyan Cao: [caoxiaoyan@snnu.edu.cn](mailto:caoxiaoyan@snnu.edu.cn).

Zhezhi Wang: [zzwang@snnu.edu.cn](mailto:zzwang@snnu.edu.cn).

**Supplementary Table 4** E/G box identidied in the promoter regions of biosynthetic genes

| Gene (length of promoter upstream ATG) | E/G-box (position upstream ATG) |
| --- | --- |
| TAT1 | CACGTT(-728);**CACGTG**(-231);CACGAC(-632);CACGTC(-641) |
| TAT2 | **CACGTG**(-1017);CACGTC(-110；-72) |
| TAT3 | CACGTT(-1134) |
| 4CL1 | 无 |
| 4CL2 | CACGTT(-414；-408);CACGTC(-322) |
| 4CL3 | CACGTT(-1419);CACGTC(-1440) |
| 4CL4 | CACGTT(-500);**CACGTG**(-509) |
| 4CL5 | CACGTA(-162) |
| 4CL7 | **CACGTG**(-232) |
| 4CL8 | **CACGTG**G(-763) |
| 4CL9 | CACGTC(-469；-274); TACGTG(-881；-90) |
| 4CL10 | CACGAC(-747) |
| HPPR1 | CACGAC(-170);**CACGTG**(-100) |
| HPPR2 | CACGTT(-1417；-1399);CACGTC(-1107);TACGTG(-985) |
| HPPR3 | CACGAC(-1092);CACGTC(-636；-83) |
| RAS1 | CACGAC(-958);TACGTG(-496) |
| RAS2 | **CACGTG**(-231) |
| RAS3 | CACGAC(-1485);CACGTT(-1129) |
| RAS4 | **CACGTG**G(-374);CACGTT(-311) |
| RAS5 | CACGTC(-128) |
| RAS6 | 无 |
| PAL1 | CACGAC(-862);CACGTT(-198);CACGTC(-631) |
| PAL2 | 无 |
| PAL3 | CACGTC(-125);CACATGG(-1133) |
| C4H1 | CACGTC(-254) |
| C4H2 | 无 |
| HPPD | CACGTC(-454);**CACGTG**(-744);TACGTG(-55) |
| CYP98A14 | **CACGTG**(-618) |
